# Lead poisoning and its effects on bone density

**DOI:** 10.1038/s41598-025-92236-w

**Published:** 2025-05-05

**Authors:** Alireza Zabihi, Omid Mehrpour, Samaneh Nakhaee, Elham Atabati

**Affiliations:** 1 Medical Toxicology and Drug Abuse Research Center (MTDRC), Birjand University of Medical Sciences, Birjand, Iran; 2 Michigan Poison & Drug Information Center, School of Medicine, Wayne State University, Detroit, MI USA; 3 Department of Rheumatology, Mashhad University of Medical Sciences, Mashhad, Iran; 4 Rheumatology Research Center, Mashhad University of Medical Sciences, Mashhad, Iran

**Keywords:** Osteoporosis, Lead poisoning, Bone density, Lead, Health care, Medical research, Risk factors

## Abstract

Lead poisoning is a significant health concern, impacting multiple organs in the body, including the musculoskeletal system. While some studies suggest a link between lead exposure and reduced Bone Mineral Density (BMD), others report no significant relationship. This study aims to address this research gap by comparing the prevalence of osteoporosis and BMD in patients with lead poisoning to that of healthy individuals. In this study, 35 lead poisoning patients admitted to the poisoning departments of Valiasr Hospital in Birjand and Imam Reza Hospital in Mashhad were compared with 35 age- and gender-matched healthy controls. The healthy group had Blood Lead concentrations (BLC), < 10 µg/dl and did not use drugs. Blood samples were collected to measure lead concentrations, and osteoporosis was compared between the two groups. Osteoporosis was diagnosed using a DEXA (Dual-energy X-ray absorptiometry) machine made in Spain. Data were entered into SPSS software version 22 and analyzed at a significance level of 5%. The mean BLC in the lead poisoning group was significantly higher than the healthy group (P < 0.05). However, there was no significant difference in the frequency distribution of osteoporosis between the groups (P > 0.05). The mean Z and T scores of femur in both groups showed no significant difference (P > 0.05). In contrast, the mean T and Z scores of L1, L2, L3, and L4 lumbar bones in the lead poisoning group were significantly lower than those in the healthy group (P < 0.05). The results of this study suggest that the prevalence of osteoporosis in people with lead poisoning is not different from that in healthy individuals. Nonetheless, bone density indices of lumbar vertebrae were affected in lead-poisoned individuals.

## Introduction

*Lead*(Pb) is a heavy metal with various applications, including the gasoline industry, residential paints, mining, smelting, casting, cosmetics, and more^1–3^. Due to its widespread use in numerous industries, lead poisoning is among the most common causes of occupational poisoning in developing countries^1^. Additionally, new forms of non-occupational lead poisoning are on the rise, as lead is present in the environment, food sources, and products such as opium and cosmetics^4–7^.

Lead poisoning affects human health, reproduction, and behavior and can even be fatal^8^. It can impact various body systems, including the digestive and nervous systems^9^and the hematopoietic, renal, hepatic, cardiovascular, respiratory, skeletal, and immune systems^10–13^.

*Osteoporosis*is a disease characterized by decreased bone density, which increases the risk of bone fractures in middle-aged and elderly individuals^14–17^. Osteoporosis is caused by a combination of non-modifiable risk factors, such as age, gender (mainly affects postmenopausal women), and reproductive history (multiparity, early menopause, and prolonged menopause), along with modifiable risk factors that include body mass index (BMI), where higher weight may increase bone density. Still, obesity may also increase marrow adiposity, compromise bone integrity, and fracture risk^16,18^. In addition, lead poisoning can be a risk factor for the development of osteoporosis. Despite numerous studies on lead poisoning, there is still substantial disagreement about its effects on bone health. Past studies have shown inconsistent results, with some suggesting a link between lead exposure and lower BMD^19–23^. Studies conducted in the general US adult population showed that BLCs were associated with lower BMD of the femur, spine^24^, lumbar spine^25^, and total BMD^26^. while some other studies find no significant relationship^27,28^An early American study concluded that higher BLCs were associated with an increased BMD in children. However, it recruited smaller samples, which may have resulted in an unreliable conclusion^29^. Another study by Li et al. found an N-shaped curve association between BLC and BMD in children and adolescents. They reported that lead exposure had different effects on BMD of different bony sites (highly cortical or trabecular regions) in childhood and adolescence. It also impacted the same bone differently among age populations and/or at different concentrations^30^. The reported inconsistencies underscore the need for further investigation. The toxic effects of lead on bone density vary among children, adolescents, and adults also in different bone sites, with additional differences among various genders, and levels of exposure^23^.

The current study is significant because it focuses on the relationship between lead poisoning and osteoporosis, which has not been consistently presented. By comparing the difference in prevalence of osteoporosis and BMD in patients with lead poisoning versus normal individuals, this study try to fill the existing gap in knowledge. Our research addresses critical questions with concerning how lead exposure affects bone health and adds to the ongoing discourse on occupational and environmental health risks of lead poisoning.

Understanding lead poisoning’s effects on bone density is important. The present study provides better insight into the potential risks of lead exposure. This study’s results contribute to improving public health policies for reducing lead exposure, particularly in vulnerable populations. By elucidating the relationship between lead poisoning and osteoporosis, we aim to inform strategies that protect high-risk groups from the long-term consequences of lead exposure.

## Materials and methods

### Study design

In this cross-sectional study, patients with lead poisoning hospitalized in the poisoning department of Valiasr Hospital in Birjand and Imam Reza Hospital in Mashhad were compared with healthy individuals. The research committee of Birjand University of Medical Sciences approved the study protocol. All methods were performed according to the relevant guidelines and regulations. The study methodology was approved by the ethics committee of Birjand University of Medical Sciences (Ir.bums.REC.1398.146).

### Inclusion and exclusion criteria

Inclusion criteria were age 20–60 years for women and 20–80 years for men, BLC above 40 µg/dl for patients in the lead poisoning group, and a BLC of less than 10 µg/dl for patients in the healthy group and having informed consent to participate in the study. Exclusion criteria included genetic bone illnesses, anticonvulsants, corticosteroid users, people with a history of smoking and substance use disorders, postmenopausal women, men with androgenic disorders, and patients with a history of cancer and chemotherapy.

### Study population

The sample size was calculated using G*Power software with an effect size *d* of 0.715, α error probability of 0.05, and power (1-β error probability) of 0.80. The number of 32 people in each group was adjusted to 35 with a probability of 10% sample dropout, and 70 people were studied. The participant enrollments were conducted during 2019 using a convenient sampling method based on the inclusion criteria. The lead poisoning group (*n* = 35) was selected from hospitalized patients in the poisoning department of Valiasr Hospital in Birjand and Imam Reza Hospital in Mashhad, and the healthy group (*n* = 35) was chosen among the employees of these two hospitals using convenience nonprobability sampling method. Participants were age- and gender-matched and were not exposed to lead at work or had a history of elevated BLCs (more than 10 µg/dl). Also, they had no history of drug abuse.

### Measurements

After obtaining informed consent, demographic information was recorded using a checklist. Blood samples (3 ml) were collected and kept on ice to measure the concentration of lead, which was assessed by atomic absorption spectrophotometry-GFAA- (Varian Co. Leicester City, UK) according to the NIOSH standard method. Graphite furnace atomic absorption spectrometry represents a sensitive, highly precise, and repeatable method for quantitatively determining lead and other heavy metals in biological fluids. A standard curve was created with at least five standard concentrations, and quality control was performed by three control samples from the Norwegian Company SERO. The Linearity threshold of the calibration curve was R^2^ > 0.99. All the measurements were duplicated and triplicated for high lead concentrations. The measurements were done by trained technicians in the same laboratory using the same equipment.

The bone mineral density (BMD) test was also performed to diagnose osteoporosis using a Dual-energy X-ray absorptiometry (DEXA) machine made in Spain at Bahar Clinic in Birjand and Imam Reza Hospital in Mashhad.

### Statistical analysis

Completed data were entered into SPSS 22 software. The Kolmogorov-Smirnov test assessed the distribution of data. An independent t-test was used to analyze quantitative data in the case of normal distribution, and the Mann-Whitney U test was used for the non-normal distribution of data. The chi-square test was used at a significance level of less than 5% to compare qualitative data. Also, the analysis involved performing linear regression to explore the relationship between various bone density scores (T and Z scores) for the femur and lumbar regions and the grouping variable.

## Results

Seventy subjects were examined, including 63 (90.0%) men and 7 (10.0%) women. There was no significant difference between the two groups regarding gender frequency distribution (*p* = 0.232). The average age of the lead poisoning and healthy groups was 50.8 ± 15.02 and 51.77 ± 14.69 years, respectively. The two studied groups were not significantly different (*P* > 0.05). The median BLC in the lead poisoning group (50 [43, 61]) was significantly higher than in the healthy group (3.4 [2.1, 5.1]) (*P* < 0.001) (Table 1). The corresponding values of mean BLCs for the two groups were 71.99 ± 101.93 µg/dl and 4.02 ± 1.92 µg/dl, respectively.

**Table 1:** Characteristics of participants in the study.

variable		Lead poisoning group (*n* = 35)	Healthy group (*n* = 35)	Total (*n* = 70)	*P*-value
Age (year)		50.8 ± 15.02	51.77 ± 14.69	51.41 ± 15.16	*P* = 0.489
Gender	Male	30 (85.7%)	33 (94.3%)	63 (90.0%)	*P* = 0.232
	Female	5 (14.3%)	2 (5.7%)	7 (10.0%)	
Lead (µg/dl)		50 [43, 61]	3.4 [2.1, 5.1]	24.2 [3.37, 50.2]	*P* < 0.001

The results are presented as Mean ± SD, frequency (percentage), or median [25th, 75th percentile]. The p-value was calculated using Chi-square statistical test, independent T-test and Mann-Whitney U test.

Table 2 shows the distribution of osteoporosis frequency in the two study groups. There were no significant differences in the frequency of osteoporosis between patients with lead poisoning and the healthy group (*P* = 0.489). Furthermore, Table 3 indicates that the mean Z and T scores of femur in both groups showed no significant difference (*P* > 0.05).

**Table 2:** Frequency distribution of osteoporosis in patients with lead poisoning compared to the healthy group.

Groups	Osteoporosis status	Frequency (percentage)	Chi-square statistical test results
Lead poisoning group	Normal	2 (5/7 %)	*P* = 0.489
	Osteopenia	12 (34/3 %)	
	Osteoporosis	21 (60 %)	
Healthy group	Normal	5 (14/3 %)	
	Osteopenia	11 (31/4 %)	
	Osteoporosis	19 (54/3 %)	

**Table 3:** Comparison of mean or median Z, T and BMD scores of the femur in the studied groups.

Variables	Groups	SD /Median (IQR) ± Mean	Result of independent t-test/ Mann-Whitney U test
Z score of femoral neck	Lead poisoning group	-0.63 ± 1.00	*P* = 0.063 df = 67.09 t = 1.406
	Healthy group	-0.20 ± 0.89	
Total Z score of femoral bone	Lead poisoning group	0.6 (-0.08, -1.3)	*P* = 0.051 Z = 1.952
	Healthy group	-0.2 (-0.8, 0.7)	
Total T score of femoral bone	Lead poisoning group	-0.98 ± 1.17	*P* = 0.086 df = 67.99 t = 1.742
	Healthy group	-0.49 ± 1.18	
T score of femoral neck	Lead poisoning group	(-1.9, -0.5)-1.4	*P* = 0.200 Z = 1.282
	Healthy group	(-0.4, -1.6) -1.0	
BMD of femur	Lead poisoning group	0.88 ± 0.18	*P* = 0.068 df = 59.90 t = 1.856
	Healthy group	0.96 ± 0.17	

The results of this study revealed that the mean T and Z scores of L1, L2, L3, and L4 lumbar bones, as well as the mean total T, Z score, and BMD score of lumbar bones in the lead poisoning group, were significantly lower than in the healthy control group (*P* < 0.05) (Table 4).

**Table 4:** Comparison of the average Z and T scores and BMD scores of lumbar bones in the studied groups.

variable	groups	SD ± Mean	Result of independent t-test/ Mann-Whitney U test
T score of L1	Lead poisoning group	-2.11 ± 1.31	*P* = 0.003 df = 66.06 t = 3.052
	Healthy group	-1.22 ± 1.10	
T score of L2	Lead poisoning group	-2.09 ± 1.53	*P* = 0.009 df = 64.42 t = 2.594
	Healthy group	-1.13 ± 1.38	
T score of L3	Lead poisoning group	-2.13 ± 1.50	*P* = 0.013 df = 65.961 t = 2.564
	Healthy group	-2.13 ± 1.50	
T score of L4	Lead poisoning group	-2.26 ± 1.72	*P* = 0.003 df = 67.994 t = 3.028
	Healthy group	-1.01 ± 1.71	
Total T score of lumbar bones	Lead poisoning group	-1.81 ± 1.25	*P* = 0.001 df = 67.666 t = 3.156
	Healthy group	-0.75 ± 1.34	
Z score of L1	Lead poisoning group	-1.6 (-2.6, -0.7)	*P* = 0.002 Z = 3.151 X^2^ = 344.500
	Healthy group	-1.2 (-1.6 , 0.1)	
Z score of L2	Lead poisoning group	-1.73 ± 1.29	*P* = 0.003 df = 65.993 t = 3.143
	Healthy group	-0.72 ± 1.38	
Z score of L3	Lead poisoning group	-1.74 ± 1.32	*P* = 0.005 df = 65.496 t = 2.896
	Healthy group	-0.73 ± 1.54	
Z score of L4	Lead poisoning group	-2.1(-3.2, -1.1)	*P* = 0.001 Z = 3.315 X^2^ = 330.500
	Healthy group	-1.2 (-1.6, 0.1)	
Total Z score of lumbar bones	Lead poisoning group	-2.20 ± 1.47	*P* = 0.002 df = 67.567 t = 3.420
	Healthy group	-1.13 ± 1.35	
BMD of lumbar spine	Lead poisoning group	0.85 ± 0.17	*P* = 0.014 df = 59.10 t = 2.537
	Healthy group	0.96 ± 0.15	

The linear regression results showed that Many T-scores, Z-scores, and BMD values (especially in the L1-L4 region) show statistically significant results. The results from Table 5 indicate that being in the case group is generally associated with lower bone density scores, particularly in the lumbar spine regions.

**Table 5:** Linear regression analysis for the T and Z scores and BMD scores of the femur and lumbar bones, using the group as the independent variable.

Variable	Coefficient	95% CI Low	95% CI High	*P*-value	*R*-squared	Intercept
T score of femoral neck	−0.948571	−2.347926	0.450784	0.180647	0.026202	−0.92
Z score of femoral neck	−0.357143	−0.798953	0.084667	0.111362	0.036854	−0.191429
Total T score of femoral bone	−0.482857	−1.02349	0.057775	0.079178	0.044626	−0.417143
total Z score of femoral bone	−0.462286	−0.942852	0.01828	0.05911	0.051402	−0.037143
TScore_L1	−0.737143	−1.315787	−0.158499	0.013304	0.086783	−1.305714
ZScore_L1	−0.794286	−1.346158	−0.242413	0.005435	0.108177	−0.902857
TScore_L2	−0.868571	−1.556759	−0.180384	0.014143	0.085319	−1.168571
ZScore_L2	−0.891429	−1.523205	−0.259652	0.006365	0.104409	−0.785714
TScore_L3	−0.902857	−1.624251	−0.181463	0.014936	0.084016	−1.18
ZScore_L3	−0.928571	−1.606289	−0.250854	0.007968	0.099042	−0.791429
TScore_L4	−1.08	−1.877809	−0.282191	0.008712	0.096909	−1.117143
ZScore_L4	−1.125714	−1.892508	−0.358921	0.004617	0.112063	−0.682857
Total T score of lumbar bones	−0.951429	−1.626098	−0.276759	0.006392	0.104306	−1.18
Total Z score of lumbar bones	−0.945714	−1.570253	−0.321176	0.003541	0.118377	−0.814286
BMD of femur	−0.074286	−0.154292	0.00572	0.068251	0.048057	0.960286
BMD of Lumbar spine	−0.096571	−0.172847	−0.020296	0.013855	0.085811	0.956286

## Discussion

This study compared bone density between patients with lead poisoning and healthy individuals. The results showed no significant difference in the frequency distribution of osteoporosis among the participants in the studied groups. In the study of Sun et al., the incidence of osteoporosis in the lead poisoning group was reported to be higher than in the control group^31^, which is inconsistent with the present study. Among the reasons for this difference is the difference in the sample size of the two studies. In the study of Sun et al., several thousand patients were examined. Also, considering that most of the patients in our study were men, the gender composition of the patients was equal in the Sun study.

Our results showed that the two groups had no differences in the femur bone’s Z, T, and BMD scores. However, the lumbar bones’ mean T and Z scores (L1, L2, L3, and L4) and the total mean T, Z, and BMD scores of the lumbar bones were significantly lower in the lead poisoning group compared to the healthy group.

Previous studies have reported conflicting results concerning the effects of lead on bone status. For instance, in line with our observation, Akbal et al.(2014) investigated BLC in male workers and found a difference between the average T score of lumbar bones (L2, L3, and L4) and the average total T score. However, they also found a significant difference between the average Z score of lumbar bones (L2, L3, and L4) and the total Z score of the two groups, so the average scores in the lead-exposed group were significantly lower than in the healthy group. Also, the BLC negatively correlated with the T and Z scores of 2–4 lumbar vertebrae and the total T and Z scores of the lumbar^32^. However, they did not report any significant differences between the mean T and Z scores of the neck and total femur bone in the case and control groups, consistent with our study’s results^32^.

Also, another study showed that the BMD of women living in polluted areas was significantly lower than women living in the control area. In addition, BMD decreased with increasing blood and urine lead concentrations in women. Low BMD was associated with high BLCs in men (OR = 4.49, 95% CI: 1.37–14.6) and higher concentrations of cadmium in women (OR = 2.5, 95% CI: 1.11–5.43)^33^. Dongre et al. (2013) examined BLC and bone mineral density in battery manufacturing workers with different lead exposure times. He included 1 to 5 years of lead exposure (mean serum lead concentration: 58.37 ± 20.2), 6 to 10 years (mean serum lead concentration: 62.52 ± 15.4), and more than 10 years of lead exposure (mean serum lead concentration: 65.5 ± 18.5). They reported a significant decrease in bone density in all three studied groups exposed to lead^34^.

In contrast, another study on 1021 individuals with environmental or occupational lead exposure did not report a significant association between BLCs and low bone density in the distal forearm^28^. A cross-sectional study on 232 premenopausal and postmenopausal individuals showed that bone mineral density is not correlated to BLCs^27^. Another study in a pediatric population showed that children with high lead exposure (mean blood lead 23.6 µg/dL) had higher BMD than children with low lead exposure. This finding may reflect a phenomenon in which lead exposure accelerates bone maturation by inhibiting the effects of parathyroid hormone-related peptides. Rapid bone maturation may lead to reduced bone density in adulthood, predisposing a person to osteoporosis in later years^29^. The different results in the previous studies may be attributed to variations in the studied populations, study designs, and sample size.

The current study has limitations, including the study design that does not allow causal inference, so conducting longitudinal studies will help clarify the relationship between lead and bone effects. The study acknowledges a limitation in the sample size and the presence of unaccounted confounding variables, potentially impacting the generalizability of the findings. Additional confounding variables such as lifestyle factors, dietary habits, and genetic predispositions could affect our results. The exclusion of these variables represents a limitation of our study. Future research should incorporate these factors to provide a more comprehensive understanding of their impact on osteoporosis indices. Also, only BLCs were measured in this study. Lead is often and easily measured in blood; however, the half-life of blood lead is short and usually indicates recent exposure. Lead is derived from different sources and stored in tissues, mainly bone, which re-enters into the blood. The results may have differed if the total body load had been better measured. Methods for detecting low concentrations of lead in bone by X-ray fluorescence can be considered in future studies. Conducting the research in limited regions may limit the generalization of our results to other populations. The investigation did not consider specific sources or duration of lead exposure, which could provide more detailed information on the relationship between lead concentrations and bone health.

## Conclusion

The results of our study indicate that the prevalence of osteoporosis in lead poisoning patients did not differ significantly from that in the healthy group. Additionally, the two groups had no significant difference in the Z and T scores and bone mineral density (BMD) in the femur bone. However, bone density indices of the lumbar vertebrae were affected in lead-poisoned individuals, with the T and Z scores of the L1-4 lumbar bones and the BMD of the lumbar bones being impacted. Consequently, lumbar and total T, Z scores in these bones were significantly lower in the lead poisoning group compared to the healthy group.

## Data Availability

All relevant data are within the manuscript.
